# Simultaneous Determination of Reserpine, Rescinnamine, and Yohimbine in Human Plasma by Ultraperformance Liquid Chromatography Tandem Mass Spectrometry

**DOI:** 10.1155/2013/940861

**Published:** 2013-12-05

**Authors:** Muzaffar Iqbal, Aftab Alam, Tanveer A. Wani, Nasr Y. Khalil

**Affiliations:** ^1^Department of Pharmaceutical Chemistry, College of Pharmacy, King Saud University, P.O. Box 2457, Riyadh 11451, Saudi Arabia; ^2^Bioavailability Laboratory, College of Pharmacy, King Saud University, Riyadh, Saudi Arabia; ^3^Department of Pharmacognosy, College of Pharmacy, Salman Bin Abdulaziz University, Alkharj, Saudi Arabia

## Abstract

A sensitive and selective UPLC-MS/MS method was developed and validated for the determination of three indolic alkaloids (reserpine, rescinnamine, and yohimbine) in human plasma using papaverine as internal standard (IS). After a one step protein precipitation with acetonitrile, separation was carried out using C18 column (50 × 2.1 mm, i.d. 1.7 **μ**m) and mobile phase consisting of acetonitrile : water : formic acid (60 : 40 : 0.1%, v/v/v) pumped at a flow rate of 0.2 mL/min. The mass spectrometric determination was carried out using an electrospray interface operated in the positive mode with multiple reaction monitoring (MRM) mode. The precursor to product ion transitions of *m/z* 609.32 > 195.01, *m/z* 635.34 > 221.03, *m/z* 355.19 > 144, and *m/z* 340.15 > 202.02 were selected for the quantification of reserpine, rescinnamine, yohimbine, and IS, respectively. The analytical response was found to be linear in the range of 0.36–400, 0.27–300, and 0.23–250 ng/mL with lower limit of quantification of 0.36, 0.27, and 0.23 ng/mL for reserpine, rescinnamine, and yohimbine, respectively. Validation was made following official guidelines. The proposed method enabled reproducible results and hence could be reliable for pharmacokinetic and toxicological analysis.

## 1. Introduction

**Figure 1:**
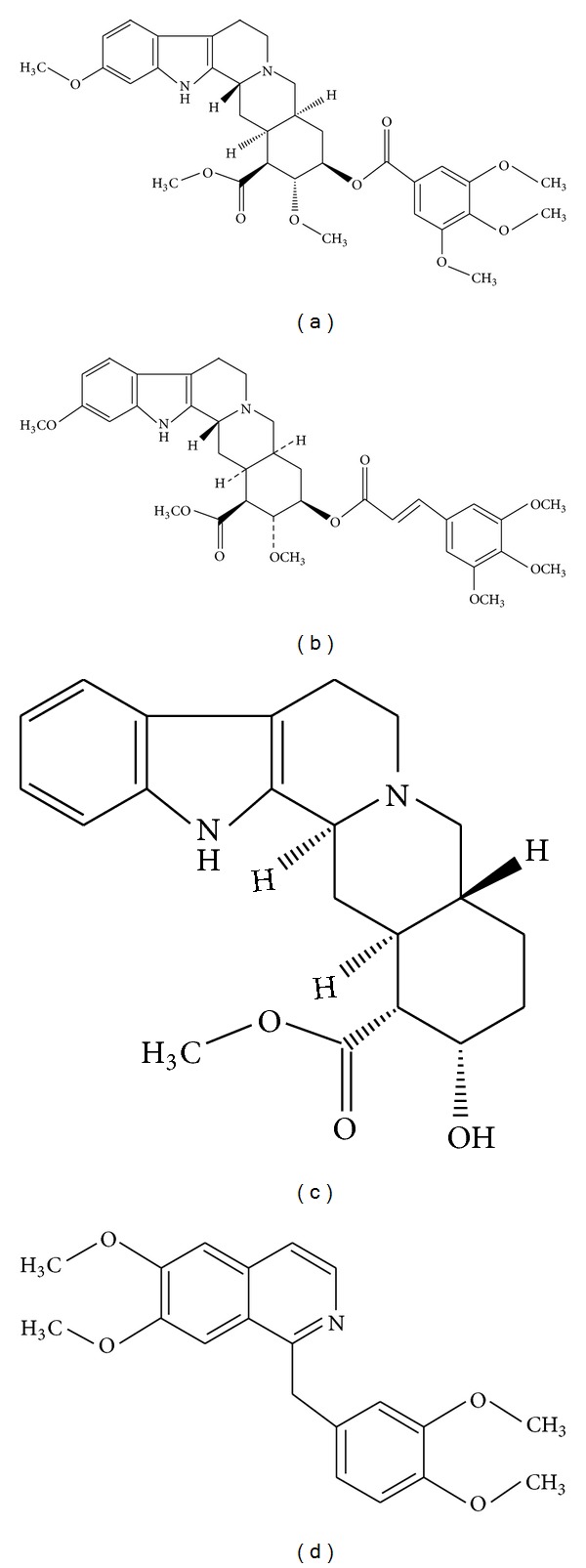
Chemical structure of reserpine (a) rescinnamine (b) yohimbine (c) and papaverine (d).

*Rauwolfia serpentina *(family: Apocynaceae) is a *medicinally important herb *commonly known as Indian snake root. It has been used for centuries in ayurvedic medicines because of their valuable therapeutic actions especially in the treatment of hypertension and psychotic disorders like schizophrenia, anxiety, insomnia, insanity, and so forth [1, 2]. *Rauwolfia serpentina *has been also used for the treatment of skin cancers, burns, eczema, and snake bite [3, 4]. *Rauvolfia verticillata *is another traditional Chinese medicinal plant that also belongs to the same Apocynaceae family and also was been used for hypertension and cardiac disorders [5]. Both *Rauwolfia serpentine* and *Rauvolfia verticillata* contains pharmacologically active indolic alkaloids, namely, reserpine, rescinnamine, and yohimbine (their chemical structures are given in Figure 1) [5, 6].

Various analytical methods have been reported for the determination of reserpine, rescinnamine, and yohimbine in their plant extracts, pharmaceutical preparation, and biological fluids. Two methods based on high-performance liquid chromatography (HPLC) have been reported for the analysis of *Rauwolfia serpentine* being one using absorption photometric detection on a photodiode array detector (to determine reserpine) [7] and the other using florescence detection (to determine both reserpine and rescinnamine) [8]. Besides, two HPLC methods with UV absorption photometric detection were also reported for the determination of yohimbine in plant bark and in commercial preparation [9] or together with reserpine in a *Rauvolfia verticillata *preparation [10]. HPTLC method for yohimbine [11] and phosphorimetric method for rescinnamine [12] and reserpine [13] were also available in literature for determination in pharmaceutical preparations. For the determination of reserpine in biological fluids, three analytical methods have been reported. The first one employed HPLC method [14] to analyze in horse plasma whereas two other employed tandem mass spectroscopy detection to analyze in equine [15] and mice [16] blood plasma. One LCMS method is also reported for the pharmacokinetic study of yohimbine in horse plasma [17].

In order to better use the therapeutic relevant herbs, it is necessary to study the pharmacokinetics of their main active components. No analytical method has been reported in the literature for the simultaneous determination of reserpine, rescinnamine, and yohimbine in biological fluids. Therefore, a method with minimal sample treatment procedure is required for the simultaneous determination of these alkaloids in plasma.

Among the currently available bioanalytical techniques, ultraperformance liquid chromatography (UPLC) has gained a considerable attention in recent years and has been emerged as the preeminent analytical tool for pharmaceutical and biomedical analysis. The van Deemter equation indicates that, as the particle size decreases to less than 2.5 *μ*m, there is a significant improvement in efficiency that will not degrade with the increasing of mobile phase flow rates. Therefore, UPLC by utilizing 1.7 *μ*m particle produces chromatographic peaks narrower than the ones obtained by conventional HPLC [18]. By using multiple reaction monitoring (MRM) as the MS detection, UPLC-MS/MS method can offer a more sensitive and selective detection, which is suitable for the pharmacokinetic study at the therapeutic dose. The present study describes a sensitive UPLC-MS/MS method using one step sample preparation process for the simultaneous determination of reserpine, rescinnamine, and yohimbine in human plasma which could be applied for the pharmacokinetic study.

## 2. Material and Methods

### 2.1. Chemicals and Reagents

Reserpine (99% purity) and yohimbine (≥98% purity) were obtained from Fluka and Merck (Germany), respectively. Rescinnamine (98%) and papaverine (IS, 99% purity, structure in Figure 1) were obtained from Acros Organics. HPLC-grade acetonitrile and methanol were obtained from Winlab Laboratory, UK. Formic acid was obtained from BDH Laboratory, England. All other reagents were of analytical grade. All aqueous solutions were prepared using water from a Milli-QR Gradient A10 water ultrapurification unit (Millipore, France).

### 2.2. Apparatus and Operating Condition

#### 2.2.1. Liquid Chromatography

The chromatography was performed on an ACQUITY UPLC system (Waters Corp., Milford, MA, USA). The UPLC system, consisted by a quaternary solvent manager, binary pump, degasser, an autosampler, and a column oven. The separation was performed on Acquity UPLC BEH C18 column (50 × 2.1 mm, i.d., 1.7 *μ*m, Waters, USA) kept at 40°C. The mobile phase consisting of a mixture of acetonitrile and water acidified with formic acid (60/40/0.1, v/v/v) pumped at a flow rate of 0.2 mL/min. The yohimbine and IS were eluted at 0.64 min, whereas reserpine and rescinnamine were eluted at 0.74 min with a total run time of 2 min. The injection volume was 5 *μ*L in partial loop mode and the temperature of the autosampler was kept at 15°C.

#### 2.2.2. Mass Spectrometric Conditions

A triple-quadrupole tandem mass spectrometer (Micromass Quattro micro Waters Corp., Milford, MA, USA) equipped with electrospray ionization (ESI) interface was used for analytical detection. The ESI source was operated in positive ionization mode. Quantification was performed using multiple reaction monitoring (MRM) mode. The transitions of *m/z* 609.32 > 195.01, *m/z* 635.34 > 221.03, *m/z* 355.19 > 144, and *m/z* 340.15 > 202.02 were selected for the quantification of reserpine, rescinnamine, yohimbine, and IS, respectively. The optimized ionization conditions were as follows: capillary voltage 3.3 kV, cone voltage 64 V, source temperature 150°C, and desolvation temperature 350°C. Nitrogen was used as desolvation and cone gas with the flow rate at 650 and 50 L/hr, respectively. Argon was used as the collision gas at a flow rate of 0.10 mL/min. The collision energies for reserpine, rescinnamine, yohimbine, and IS were 48 eV, 34 eV, 30 eV, and 28 eV, respectively, with the dwell time of 0.077 s. The Mass Lynx software (Version 4.1, SCN 714) was used to control the UPLC-MS/MS system and data was collected and processed using TargetLynx program.

### 2.3. Calibration Standards and Quality Control Samples

A standard stock solution of reserpine, rescinnamine, yohimbine, and IS was prepared by dissolving in methanol to give a final concentration of 500 *μ*g/mL. The solutions were kept in the refrigerator and could be used for 15 days from the date of preparation. Stock solution of reserpine, rescinnamine, and yohimbine was used for calibration standards and quality control (QC) samples, respectively. The standard stock solutions of reserpine, rescinnamine, and yohimbine was then serially diluted in methanol : water (50 : 50%, v/v) to provide standard working solution in the following concentration range: 3.6 to 4000 ng/mL (reserpine), 2.7 to 3000 ng/mL (rescinnamine), and 2.3 to 2500 ng/mL (yohimbine). A 20 *μ*L aliquot of each working solutions was added to blank human plasma to yield effective calibration standards ranging from 0.36 to 400 ng/mL (reserpine), 0.27 to 300 ng/mL (rescinnamine), and 0.23 to 250 ng/mL (yohimbine). The effective plasma concentrations of low (LQC), medium (MQC), and high (HQC) QC samples were prepared in a similar way for reserpine (0.96, 24, and 300 ng/mL), rescinnamine (0.80, 20, and 250 ng/mL), and yohimbine (0.64, 16, and 200 ng/mL), respectively. Analytes spiked plasma used for calibration standards and quality control samples were kept at −80°C until assayed or used for validating the assay procedures. The IS working solution (1 *μ*g/mL) for routine use was prepared by diluting the papaverine stock solution in methanol : water (50 : 50%, v/v).

### 2.4. Sample Preparation

Plasma samples stored at around −80°C were thawed, left for 1 h, and vortex for 30 s at room-temperature before extraction to ensure homogeneity. To 200 *μ*L of blank plasma sample 20 *μ*L of working standard solution and 10 *μ*L (1 *μ*g/mL) of IS (except blank sample) were added. The samples were vortex-mixed for about 30 s and 770 *μ*L of acetonitrile was added. The samples were again vortex mixed gently for 1.5 min and then cold-centrifuged for 12 min at 10000 rpm. After centrifugation, 600 *μ*L of supernatant was transferred into HPLC vial, and 5 *μ*L volumes (in partial loop with needle over fill mode) of the sample were subjected to the analysis by UPLC-MS/MS.

### 2.5. Method Validation

A full method validation was performed according to guidelines set by the United States Food and Drug Administration (US-FDA) and European Medicines Agency (EMEA) guidelines [19, 20]. The validation of this procedure was performed in human plasma in order to evaluate the method in terms of selectivity, linearity of response, accuracy, precision, recovery, and stability of analytes during both short-term sample processing and long-term storage.

#### 2.5.1. Selectivity and Specificity

The selectivity of the method towards endogenous plasma matrix components, metabolites, and component medications were assessed in human plasma. Among the analyzed batch of samples, human plasma samples showing not significant interference at the retention time of analytes (LLOQ level) and IS (50 ng/mL) were selected as calibration matrix and QC matrix. These samples were analyzed and no relevant interferences were found at the retention time of the analytes and papaverine.

#### 2.5.2. Linear Response and Limit of Quantification

The linear response of the method was determined by the evaluation of eight points analytical curve: 0.36 to 400 ng/mL (reserpine) 0.27 to 300 ng/mL (rescinnamine), and 0.23 to 250 ng/mL (yohimbine). Analytical curves from accepted three precision and accuracy validation method were used to establish linearity. Analytical curves were plotted using a least square linear regression model *y* = *mx* + *b*, weighted by 1/*x* ^2^, in which *y* is the peak area ratio (analyte/IS), *m* is slope of the analytical curve, *b* is the *y*-axis intercept of the analytical curve, and *x* is the analyte (reserpine, rescinnamine, and yohimbine) concentration. The concentrations of reserpine, rescinnamine, and yohimbine were calculated from each analytical curve and the resulting calculated parameters were used to determine concentrations of these analytes in quality control samples. The determination coefficient *R* ^2^ > 0.98 was desirable for all the analytical curves. The lowest concentration on the analytical curve was to be accepted as the lower limit of quantification (LLOQ), if the analyte response was at least five times more than that of drug free (blank) extracted plasma and highest concentration was to be accepted as upper limit of quantification (ULOQ). In addition, the analyte peak of LLOQ sample should be identifiable, discrete, and reproducible with accuracy within ±20% and a precision ≤20%. The deviation of standards other than LLOQ from the nominal concentration should not be more than ±15.0%.

#### 2.5.3. Precision and Accuracy

Precision of an analytical method describes the closeness of two or more measurements to each other whereas the accuracy of an analytical method describes the closeness of mean test results obtained by the method to the true value (concentration) of the analyte. Intra- and interday accuracies are expressed as a percentage of deviation from the respective nominal value. The precision of the assay was measured by the percent coefficient of variation (%CV) at three different concentrations in human plasma. Intraday precision and accuracy were assessed by analyzing six replicates of the quality control samples at three different levels (reserpine (0.96, 24, and 300 ng/mL), rescinnamine (0.80, 20, and 250 ng/mL), and yohimbine (0.64, 16, and 200 ng/mL)) during a single analytical run. Similarly the interday precision and accuracy were assessed by analyzing 18 replicates of the quality control samples at different concentration levels through three precision and accuracy batches runs on 3 consecutive validation days. The deviation at each concentration level from the nominal concentration was expected to be ≤15.0% for precision whereas the mean accuracy should not deviate by ±15.0%.

#### 2.5.4. Extraction Recovery and Matrix Effect

The recoveries of reserpine, rescinnamine, and yohimbine at three QC levels were determined by comparing peak area ratios of the indolic alkaloid in analyte spiked plasma samples before and after proceeding the extraction of the added analytes. Calculation of the matrix effects was conducted by comparing the ratio of the indolic alkaloid signals obtained from analyte spiked plasma with the one obtained from aqueous solution with the same concentration of the indolic alkaloids. Deviation in analyte concentration of a maximum of 15% was considered acceptable and is in line with current EMEA guideline [19]. The extraction recovery and matrix effects of IS were also simultaneously evaluated using the same method.

#### 2.5.5. Stability

Stability of reserpine, rescinnamine, and yohimbine in plasma was assessed by analyzing six replicates of QC samples at low and high concentrations under a variety of storage and processing conditions. Bench-top stability was assessed after exposure of the plasma samples to room temperature for *∼*6 h, which exceeds the residence time of the sample processing procedures. The freeze-thaw stability was evaluated after undergoing three freeze (at around −80°C)-thaw (room temperature) cycles. The processed sample stability was determined by storing the reconstituted QC samples for *∼*48 h under autosampler condition (maintained at 15°C) before being analyzed. Long-term stability was assessed after storage of the test samples at around −80°C for 30 days. The samples were considered stable in plasma at each concentration if the deviation from the mean calculated concentration of stability quality control samples was within ±15%.

## 3. Results and Discussion

### 3.1. Optimization of Chromatographic Condition

Initial feasibility experiments of various mixtures of organic solvents such as acetonitrile and methanol along with pure water, both having 0.1% formic acid along with altered flow-rates (in the range of 0.20–0.4 mL/min), were performed to optimize an effective chromatographic resolution of reserpine, rescinnamine, yohimbine, and IS. Mobile phase containing methanol and water with 0.1% formic acid did not produce satisfactory separation results. Then acetonitrile with water having 0.1% formic acid was tried starting from 50 : 50 ratio with altered flow rate in range from 0.2 to 0.4 mL/min. Results was found to be satisfactory and the best resolution of peaks was achieved with an isocratic elution by a mobile phase comprising acetonitrile : water : formic acid (60 : 40 : 0.1,v/v/v) at a flow rate of 0.2 mL/min, on a C18 column (50 × 2.1 mm, i.d. 1.7 *μ*m). The selected conditions were found to be suitable for the adequate analytes response using electrospray.

**Figure 2:**
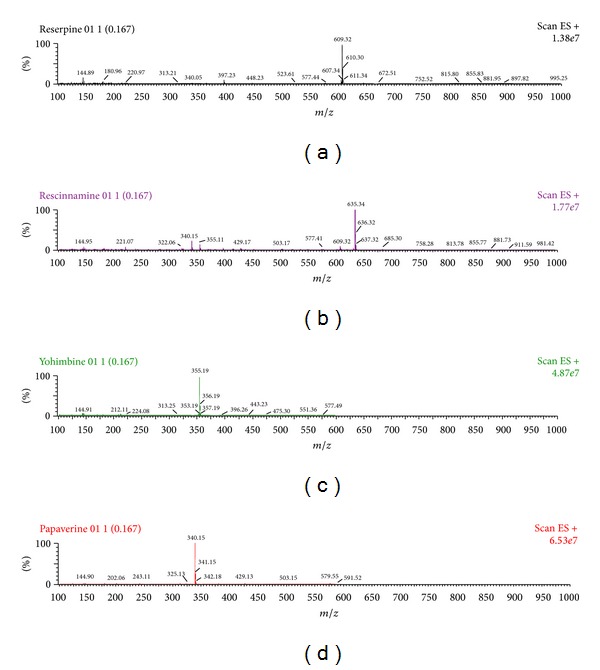
The MS spectra (parent ion) of reserpine (a), rescinnamine (b) yohimbine (c) and papaverine (d).

**Figure 3:**
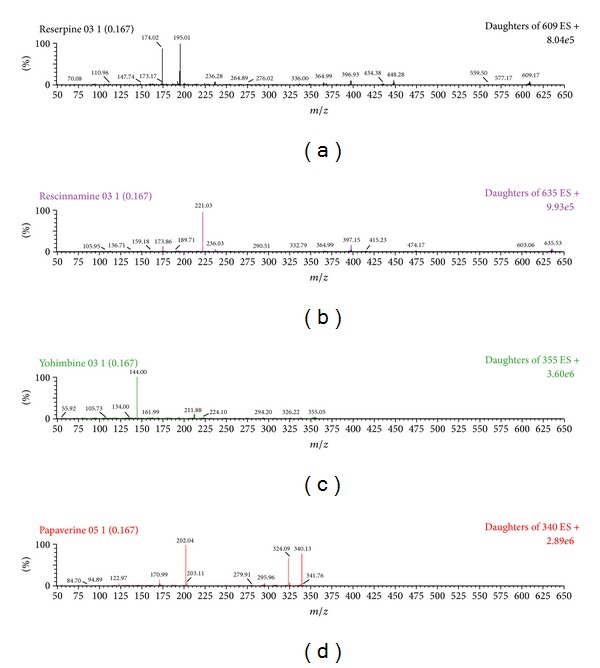
The MS/MS spectra (daughter ion) of reserpine (a), rescinnamine (b) yohimbine (c) and papaverine (d).

UPLC-MS/MS operation parameters were carefully optimized for the determination of indole alkaloids and IS. A standard solution (200 ng/mL) of reserpine, rescinnamine, yohimbine, and IS was directly infused along with the mobile phase into the mass spectrometer with ESI as the ionization source. The mass spectrometer was tuned initially in both positive and negative ionization modes for the indole alkaloids and papaverine. It was observed that the signal intensities of positive ions were much more intense than those of negative ions. Parameters, such as capillary and cone voltage, desolvation temperature, ESI source temperature, and flow rate of desolvation gas and cone gas, were optimized to obtain the optimum intensity of deprotonated molecules of indole alkaloids and papaverine for quantification. Among the parameters, capillary voltage and especially the cone voltage were important parameters. The precursor ion intensities increased significantly when cone voltage was gradually increased. Lastly, analytes produced the strongest ion signals when cone voltage was set up at 64 V. While cone voltage exceeded 64 V, the ion signals decreased rapidly. The collision energy was investigated from 10 to 50 eV to optimize the response of product ion, and the best values were selected for each product ions. The MS spectra (parent ion) and MS/MS spectra (daughter ion) for reserpine, rescinnamine, yohimbine, and papaverine are shown in Figures 2 and 3, respectively.

### 3.2. Optimization of Sample Processing

Protein precipitation was used for sample preparation in this study. Protein precipitation can be helpful in producing a clean sample and avoiding endogenous substances in plasma with the analytes and IS onto the column and MS system. Clean samples are essential for minimizing ion suppression and matrix effect in UPLC-MS/MS analysis. Two organic solvents, methanol and acetonitrile, in presence or absence of formic acid were evaluated. Finally methanol was found to be optimal, which can produce a clean chromatogram for a blank plasma sample and yield the highest recovery for the analytes from the human plasma.

### 3.3. Method Validation

#### 3.3.1. Selectivity

**Figure 4:**
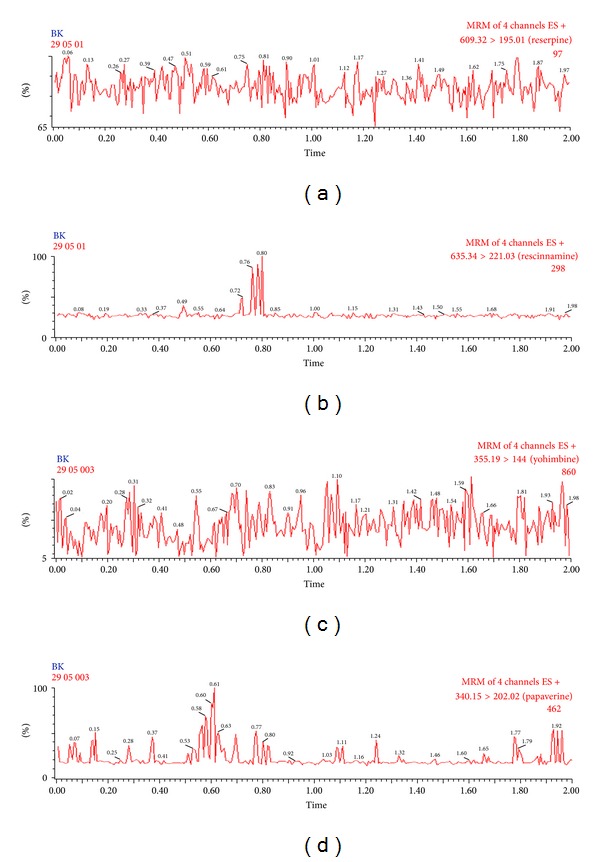
Representative MRM chromatograms of extracted blank plasma sample of reserpine (a), rescinnamine (b) yohimbine (c) and papaverine (d).

**Figure 5:**
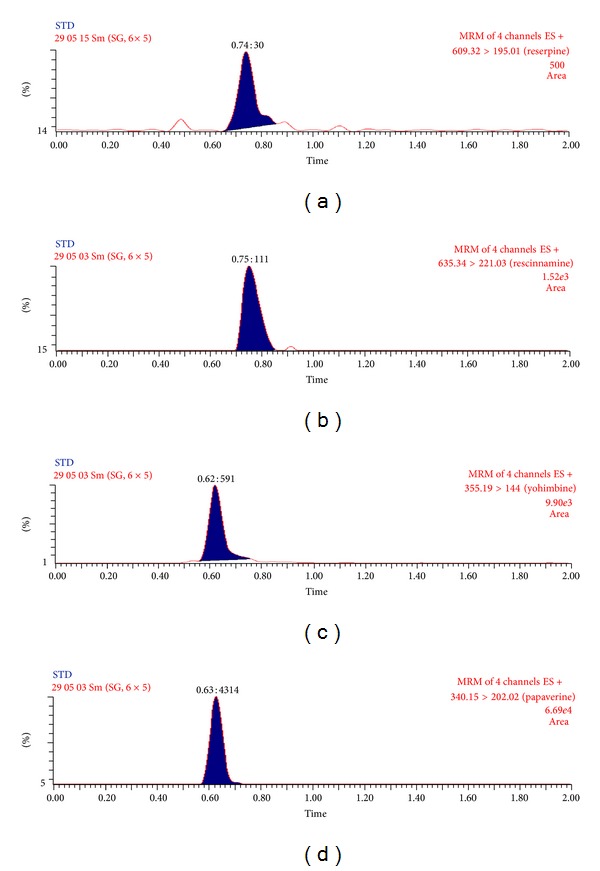
Representative MRM chromatograms at LLOQ level of reserpine (a), rescinnamine (b) yohimbine (c) and papaverine (d).

**Figure 6:**
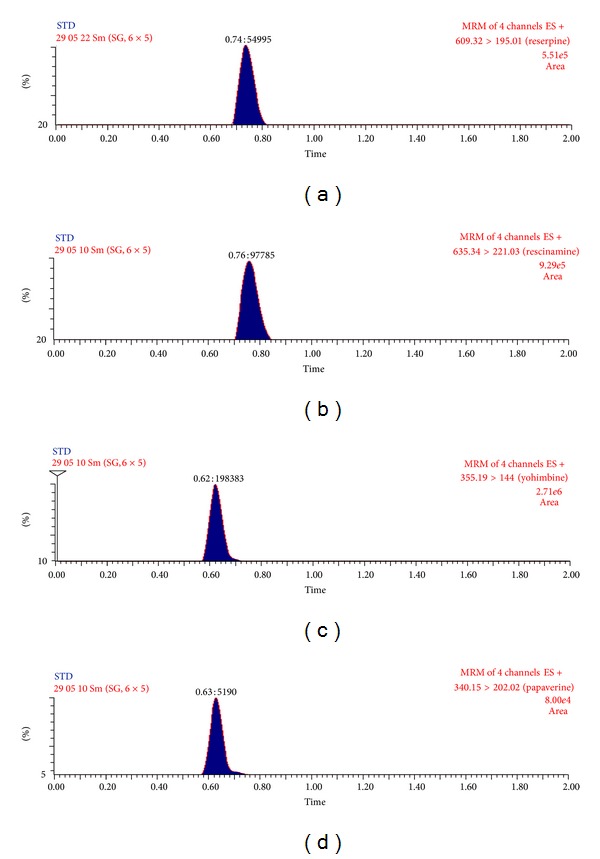
Representative MRM chromatograms at ULOQ level of reserpine (a), rescinnamine (b) yohimbine (c) and papaverine (d).

Selectivity of the method was assessed by comparing thechromatograms of human blank plasma with the corresponding spiked LLOQ sample. Only those lots which are under the acceptance criteria (<20% in comparison to the spiked LLOQ and <5% in comparison to IS area) were selected. This infers that there were no potential significant endogenous substances in plasma that interfered with the peaks of analyte and IS. Thus the method seems to be selective enough for determination of reserpine, rescinnamine, yohimbine, and IS in plasma. Representative MRM chromatograms obtained from blank plasma showing no interference at the retention time of analytes and IS are shown in Figure 4. Representative chromatograms at LLOQ and ULOQ level are shown in Figures 5 and 6, respectively.

#### 3.3.2. Linear Response and Limit of Quantification

The linearity of the method was determined by a weighted least square regression analysis of standard plot associated with an eight-point standard curve. The analytical curves were generated by plotting area ratio (analytes/IS) as a function of analyte concentration. Analytical curve was found to be linear in range of 0.36–400, 0.27–300, and 0.23–250 ng/mL for reserpine, rescinnamine, and yohimbine, respectively, in human plasma. The determination coefficients (*R* ^2^) were consistently greater than 0.995 during the course of validation. The LLOQs value for this assay was 0.36, 0.27, and 0.23 ng/mL for reserpine, rescinnamine, and yohimbine, respectively, in plasma. Representative LLOQ is sensitive enough to investigate the pharmacokinetic behavior of these analytes in plasma sample.

#### 3.3.3. Precision and Accuracy

Table 1 summarizes the inter- and intraday precision and accuracy values for QC samples in human plasma. For reserpine, the intra- and interday precisions were ≤8.3, whereas the intra- and interday accuracies were in the range of 89.8–111.1%. For rescinnamine, the intra- and interday precisions were ≤9.6, whereas the intra- and interday accuracies were in the range of 91.2–108.1%. Similarly, for yohimbine, the intra- and interday precisions were ≤9.8, whereas the intra- and interday accuracies were in the range of 91.1–110.4%. These results indicate that the method has good precision and accuracy was also adequate for the level of analyte concentration in the samples and are within the acceptance limit of ≤15% and ±<15% for precision and accuracy, respectively [19, 20].

#### 3.3.4. Recovery and Matrix Effects

The percentage recoveries (mean ± SD) of reserpine, rescinnamine, and yohimbine obtained from plasma at three different concentration levels (reserpine (0.96, 24, and 300 ng/mL), rescinnamine (0.80, 20, and 250 ng/mL), and yohimbine (0.64, 16, and 200 ng/mL)) were 69.3 to 72.9%, 64.9 to 81.7%, and 69.8 to 75.8%, respectively. The mean recovery for the papaverine (IS) at the concentration employed was 76.9%. This result indicates that the extraction efficiency for reserpine, rescinnamine, and yohimbine using protein precipitation method was satisfactory, consistent, and concentration independent. The ionization suppression or enhancement effects were between ±15%, indicating no relative matrix effects.

#### 3.3.5. Stability

The stabilities of reserpine, rescinnamine, and yohimbine were investigated at two concentrations of QC samples (low and high concentrations) to cover expected conditions during analysis, storage, and processing of all samples, which include the stability data from various stability exercises like bench-top, freeze/thaw, processed sample, and long-term stability tests. The stability results summarized in Table 2 indicate that reserpine, rescinnamine, and yohimbine spiked into human plasma were stable for at least 6.0 h at room temperature, for at least 48 h in final extract at 15°C under autosampler storage condition and during three freeze-thaw cycles when stored at around −80°C and thawed to room temperature.

## 4. Conclusions 

An efficient, reliable, and novel UPLC-MS/MS method was successfully developed for the simultaneous determination of indolic alkaloids (reserpine, rescinnamine, and yohimbine) in human plasma sample. The developed method is validated according to official guideline. Proposed method is advantageous in term of sensitivity, one step sample preparation, and short runtime (2.0 min) and hence suitable for high-throughput analysis. The analytical method presented here will be useful for the quantitative investigation of these indolic alkaloids in pharmacokinetic and toxicological analysis.

## Figures and Tables

**Table 1:** Intra- and inter-day precision and accuracy of reserpine, rescinnamine and yohimbine in plasma.

Analyte	Nominal concentration (ng/mL)	Intraday variation (*n* = 6)			Inter-day variation (*n* = 18)		
		Measured conc. (ng/mL ± SD)	Precision (CV, %)	Accuracy (recovery, %)	Measured conc. (ng/mL ± SD)	Precision (CV, %)	Accuracy (recovery, %)
Reserpine	0.96	1.08 ± 0.04	3.7	112.5	1.07 ± 0.06	6.0	111.1
	24	23.9 ± 1.99	8.3	99.7	22.9 ± 1.76	7.6	95.6
	300	269.3 ± 13.4	4.9	89.8	272.4 ± 17.6	6.4	111.8
Rescinnamine	0.80	0.87 ± 0.04	5.0	108.1	0.86 ± 0.05	5.4	108.1
	20	19.3 ± 1.86	9.6	96.7	20.54 ± 1.91	9.3	102.7
	250	228.0 ± 9.9	4.3	91.2	238.0 ± 16.78	7.0	95.2
Yohimbine	0.64	0.71 ± 0.04	5.4	110.4	0.69 ± 1.03	4.6	108.1
	16	17.0 ± 1.67	9.8	106.3	17.06 ± 1.43	8.4	106.6
	200	182.2 ± 5.1	2.7	91.1	183.39 ± 6.71	3.6	91.2

**Table 2:** Stability data of reserpine, rescinnamine and yohimbine in plasma.

Analyte	Nominal conc. (ng/mL)	Bench top stability (*n* = 6)			Freeze-thaw (*n* = 6)			Processed sample (*n* = 6)		
		Measured conc. (ng/mL ± SD)	Precision (CV, %)	Accuracy (%)	Measured conc. (ng/mL ± SD)	Precision (CV, %)	Accuracy (%)	Measured conc. (ng/mL ± SD)	Precision (CV, %)	Accuracy (%)
Reserpine	0.96	1.03 ± 0.07	6.7	116.9	1.06 ± 0.04	3.5	110.6	0.98 ± 0.06	5.9	101.2
	300	277.1 ± 18	6.7	92.4	217.8 ± 23.5	8.7	90.6	279.4 ± 4.3	1.6	93.0
Rescinnamine	0.80	0.87 ± 0.02	2.8	108.1	0.82 ± 0.04	5.4	102.08	0.82 ± 0.04	5.2	102.3
	250	229.0 ± 11	4.7	91.6	242.8 ± 8.04	3.3	97.1	241.7 ± 11	4.9	96.7
Yohimbine	0.64	0.70 ± 0.08	12.0	109.3	0.69 ± 0.03	4.4	107.5	0.64 ± 0.05	7.8	100.5
	200	197.0 ± 8.0	3.8	98.4	203.3 ± 8.8	4.4	101.7	196.2 ± 7.9	4.1	98.1
